# Cytomegalovirus enterocolitis in a patient with dihydropyrimidine dehydrogenase deficiency after capecitabine treatment: A case report

**DOI:** 10.1016/j.ijscr.2019.02.022

**Published:** 2019-02-23

**Authors:** Fumiya Inoue, Takuya Yano, Masahiro Nakahara, Hiroshi Okuda, Hironobu Amano, Shuji Yonehara, Toshio Noriyuki

**Affiliations:** a Department of Surgery, Onomichi General Hospital, 1-10-23 Hirahara, Onomichi-shi, Hiroshima, 722-8508, Japan; b Department of Pathology, Onomichi General Hospital, 1-10-23 Hirahara, Onomichi-shi, Hiroshima, 722-8508, Japan

**Keywords:** 5-FU, 5-fluorouracil, DPD, dihydropyrimidine dehydrogenase, PBMCs, peripheral blood mononuclear cells, CMV, cytomegalovirus, MRSA, methicillin-resistant *Staphylococcus aureus*, PCR, polymerase chain reaction, *DPYD*, dihydropyrimidine dehydrogenase, DPD deficiency, 5-Fluorouracil, Cytomegalovirus, Case report

## Abstract

**Introduction:**

5-Fluorouracil (5-FU) is widely used for cancer treatment. The reduced activity of dihydropyrimidine dehydrogenase (DPD), the key enzyme in 5-FU inactivation, increases a patient’s risk of developing severe 5-FU related toxicity. However, screening for DPD deficiency is rarely performed before 5-FU administration.

**Presentation of case:**

Our patient was a 69-year-old man with rectal cancer (T2N1bM0 stage IIIA) who underwent laparoscopic low anterior resection. He developed severe neutropenia and diarrhea 15 days after the administration of capecitabine for adjuvant chemotherapy, and was admitted to our hospital. Four days after admission, he was transferred to the intensive care unit for sepsis. DPD protein screening revealed DPD deficiency. On day 27, massive melena suddenly appeared. He died of continual bleeding 41 days after admission. Pathological autopsy revealed cytomegalovirus enterocolitis.

**Discussion:**

The administration of 5-FU to patients with DPD deficiency is lethal. Genotypic and phenotypic assessments are reliable tests for DPD deficiency. A genetic study can effectively screen for DPD deficiency; however, its use has not been established in the national insurance system. Patients with DPD deficiency tend to develop severe neutropenia, so clinicians should pay attention to opportunistic infections such as cytomegalovirus enterocolitis.

**Conclusion:**

Screening for DPD deficiency is necessary prior to 5-FU administration.

## Highlights

• In the patients with DPD deficiency, advert events appear more rapidly than usual and can be lethal.
• Cytomegalovirus enterocolitis is likely to occur in the state of immunosuppression and lethal because of massive bleeding or perforation.
• Screening for DPD deficiency should be done, and genetic study can be effective screening for DPD deficiency.

## 1 Introduction

5-Fluorouracil (5-FU) has been widely used for almost 50 years for the treatment of cancers of the gastrointestinal tract, breast, head, and neck. DPD is the initial and rate-limiting enzyme of 5-FU catabolism, which occurs mainly in the liver; thus, DPD deficiency is associated with severe 5-FU toxicity, including neutropenia, mucositis, and diarrhea [1]. A correlation has been observed between the pretreatment activity of DPD in peripheral blood mononuclear cells (PBMCs) and the liver; DPD deficiency can be diagnosed by measuring DPD protein in the PBMCs isolated from blood samples [2].

In DPD deficiency, it is necessary to be aware of infectious enteritis and other opportunistic infections because of neutropenia. Cytomegalovirus (CMV) is a major opportunistic pathogen of gastrointestinal diseases in immunosuppressed patients. CMV enterocolitis is lethal because it can result in massive bleeding and gastrointestinal perforation [3]. To the best of our knowledge, only one case report of CMV enterocolitis with a partial DPD deficiency has been reported [4]. Here we report a thought-provoking case of CMV enterocolitis with partial DPD deficiency during neoadjuvant chemotherapy. This study is reported in line with SCARE criteria [5].

## 2 Case presentation

**Fig. 1:**
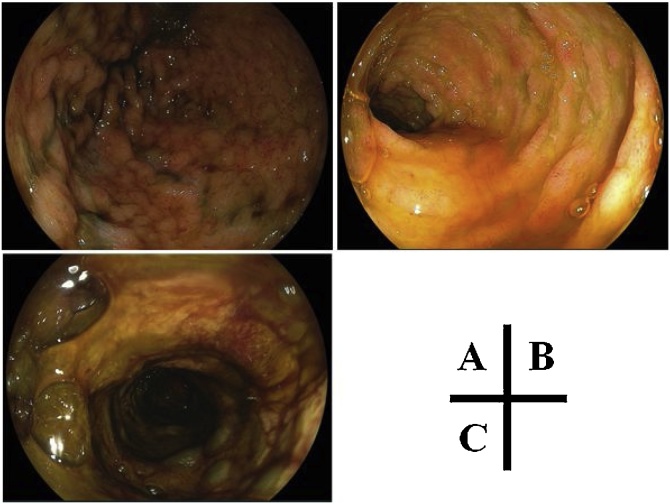
Upper and lower gastrointestinal endoscopy revealing multiple erosions and ulcers in the stomach (A), duodenum (B), and rectum (C).

**Fig. 2:**
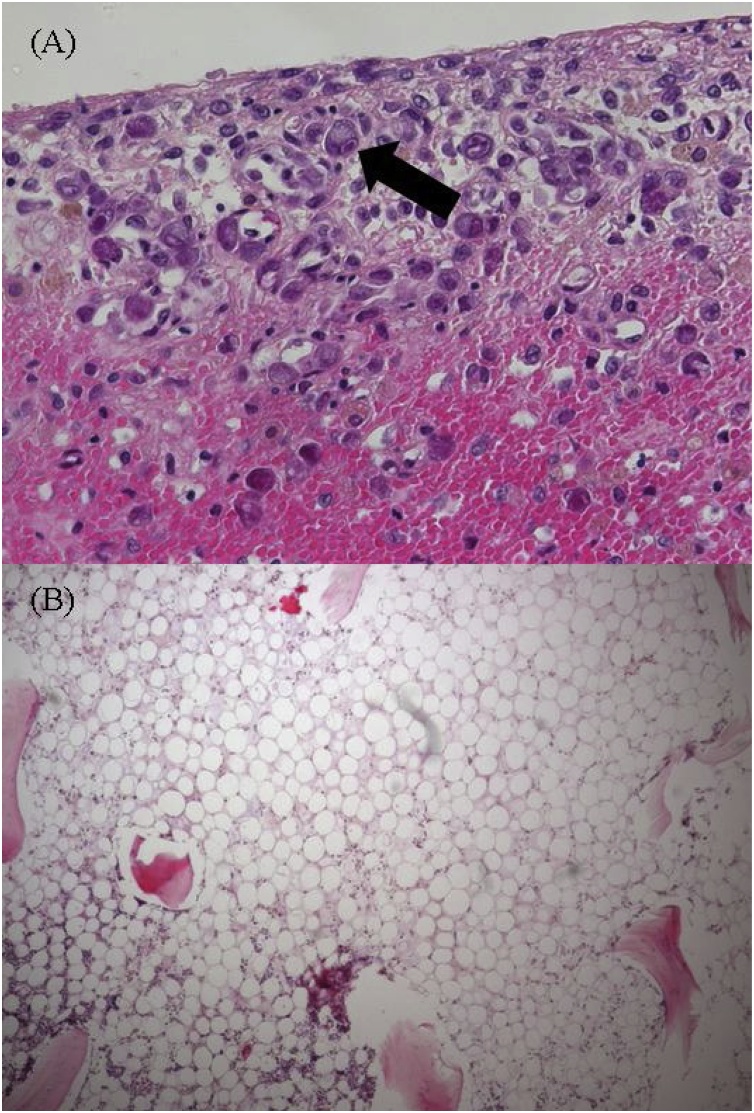
A) Cytomegalovirus-infected cells with intranuclear inclusion (black arrow) in the mucosa of the rectum (H＆E, original magnification, ×400). B) Hypoplasia of the bone marrow (H＆E, original magnification, ×100). H&E, hematoxylin and eosin.

A 69-year-old man with no medical history underwent laparoscopic low anterior resection for rectal cancer (T2N1bM0 stage IIIA), followed by adjuvant chemotherapy consisting of capecitabine 3600 mg/day on 36 days after surgery. Fifteen days post-administration, he was hospitalized with severe diarrhea, melena, fever, and neutropenia. A thoraco-abdominopelvic computed tomography scan showed an edematous small intestine; thus, the capecitabine was stopped and the antibiotic cefmetazole was started. On day 4, because of clinical worsening with low blood pressure and a decreased level of consciousness, he was transferred to the intensive care unit with sepsis and multiorgan failure. Laboratory tests showed bicytopenia (neutrophil count, 16/μL; platelet count, 4,4000/μL), coagulopathy (prothrombin time, 32%), metabolic acidosis (pH 7.19), hyperlactatemia (9.7 mmol/L), and renal failure (plasma creatinine, 2.7 mg/dL). Broad-spectrum anti-infectious treatment (meropenem, caspofungin) was started concomitantly with the administration of granulocyte-colony stimulating factor, vasopressors, and continuous hemodiafiltration. On day 7, pneumonia was evident on a chest X-ray, and a sputum culture was positive for methicillin-resistant *Staphylococcus aureus* (MRSA); thus, the additional administration of vancomycin was started. On 13 day, blood and stool cultures were positive for MRSA. On day 27, massive melena suddenly appeared, and upper and lower gastrointestinal endoscopy showed severe ulcers in the stomach (Fig. 1), duodenum, and rectum. DPD protein quantification in the PMBC was 17.1 U/mg (normal range, 33.6–183.6 U/mg in PBMC). The continual massive bleeding gradually deteriorated the patient’s hemodynamic state, and he died on day 41. A pathological autopsy revealed many intracellular inclusions from the jejunum to the rectum, indicating CMV enterocolitis and bone marrow hypoplasia (Fig. 2).

## 3 Discussion

The pyrimidine analog 5-FU and its oral pro-drug capecitabine are the most commonly prescribed anti-cancer chemotherapeutic agents. DPD catabolizes ˜80% of the administered dose of 5-FU, and its activity is highly variable throughout the population [6]. The two most reliable tests for predicting 5-FU toxicity are genotypic and phenotypic assessments (DPD activity in PBMC, uracil breath test, endogenous plasma/urine uracil/dihydrouracil, sampling pharmacokinetics model after 5-FU test dose [7]). A previous study showed that patients with DPD activity < 70% tend to develop severe 5-FU–associated adverse event [1]. 5-FU can cause toxicity in several body systems (e.g., gastrointestinal, hematological, neurological). Gastrointestinal symptoms are the major adverse events of 5-FU. Gastroenteritis in patients with 5-FU is caused by toxicity of itself, but patients are highly susceptible to infectious gastroenteritis in 5-FU–induced neutropenia. A significantly higher percentage (55%) of patients with decreased DPD activity suffered from grade IV neutropenia than patients with normal DPD activity (13%) [1]. Therefore, it is important to pay attention to the presence of infectious enteritis and other opportunistic infections in patients with DPD deficiency.

In this case, severe adverse events such as neutropenia and diarrhea appeared 15 days after the administration of capecitabine. We found DPD deficiency as a cause of the rapid time to toxicity. Also, all five patients with DPD deficiency who developed fatal gastrointestinal disease (Table 1) suffered from adverse events for a month [[8], [9], [10]]. Oral dosing of uridine triacetate (Vistogard) within 96 h for the emergency treatment of patients following 5-FU or capecitabine overdose improves survival dramatically [11]. Therefore, uridine triacetate may be a useful treatment for adverse events related to DPD deficiency because of its rapid time to toxicity.

**Table 1:** Five cases of fatal gastrointestinal disease in patients with DPD deficiency.

Age	Sex	Location	Chemotherapy	Onset day from infusion	Management	Dead day from infusion	Cause of death	Genotypic study	DPD activity
75	male	sigmoid	FOLFOX	2 days	amikacin and imipenem	10 days	septic shock	homozygote genotype for DPYD*2A	Not performed
73	male	sigmoid colon	5-FU and leucovorin	12 days	unknown	16 days	unknown	heterozygote genotype for DPYD*2A	Not performed
58	female	sigmoid colon	5-FU and leucovorin	7 days	systemic antibiotics and hemodynamic support	5 weeks	sepsis, ARDS, hypotension	heterozygote genotype for DPYD*2A	Not performed
53	female	rectum	5-FU	1 days	intravenous hydration	1 week	metabolic encephalopathy, difficult control arrhythmia	homozygote genotype for DPYD*2A	0.06 nmol/mg/min^a^
44	female	rectum	5-FU and leucovorin	5 days	transfusion with erythrocytes and thrombocytes	13 days	infectious complications	homozygote genotype for DPYD*2A	0.09 nmol/mg/h^b^

DPD, dihydropyrimidine dehydrogenase; *DPYD*, dihydropyrimidine dehydrogenase; 5-FU, 5-Fluorouracil; FOLFOX, fluorouracil leucovorin, and oxaliplatin.

a Normal DPD enzyme activity in PBMC was above 0.064 nmol/mg/min in this report.

b The same tests showed a DPD level of 10.0 ± 3.4 nmol/mg/h in PBMC from 22 healthy individuals in this report.

We could not diagnose the cause of gastroenterocolitis and our patient died of massive bleeding on 41 day after admission. A pathological autopsy revealed CMV enterocolitis as the cause of the massive bleeding. CMV enterocolitis is a lethal organ disease because it can result in massive bleeding and perforation [3]. Therefore, an early-stage diagnosis is essential, but it is difficult to make definitively. A histopathological diagnosis is the gold standard for diagnosis (by identification of CMV inclusion bodies), but endoscopic biopsy is invasive and carries the risk of hemorrhage or perforation [12]. In this case, we could not perform a biopsy due to the perforation concern. The CMV blood antigenemia assay is a noninvasive method for detecting CMV viremia, but its sensitivity in CMV–gastrointestinal disease is not high (47–75%) [12,13]. The detection of CMV DNA through polymerase chain reaction (PCR) in tissue biopsies and the blood is the most useful technique for diagnosing CMV infection and disease. The advantages of PCR are its rapid turnaround and high sensitivity [14]. We should have performed PCR or an antigenemia assay and administered antiviral drugs empirically in this case. Among the five patients with DPD deficiency and fatal gastrointestinal disease, neither PCR nor an antigenemia assay was performed (Table 1). We should keep in mind the possibility of opportunistic infections in patients with DPD deficiency.

Functional dihydropyrimidine dehydrogenase (*DPYD*) gene variants were recently found to be associated with reduced/abrogated DPD activity [6]. To date, three DPYD genetic variants have been identified as being consistently associated with 5-FU risk of toxicity: *2 A rs3918290 G > A, *13 rs55886062 T > G, and rs67376798 A > T. The *2 A rs3918290 G > A mutation is commonly correlated with the time to toxicity of 5-FU [15]. Among the five patients with DPD deficiency and fatal gastrointestinal disease (Table 1), all had the *2 A rs3918290 G > A variant. Unfortunately, we could not perform a genetic assessment. In the past, screening for DPD deficiency was considered unrealistic in the terms of cost, but available analyses suggest that DPYD genotype–guided dosing might significantly improve 5-FU therapy safety and cost [16,17]. A prospective study showed that screening for only DPYD*2 A improved safety and saved cost for 5-FU therapy and that patients who are DPYD*2 A variant allele carriers can be treated with starting doses reduced by 50% [18]. There is a limitation of screening only for DPYD*2 A because its frequency was only 0.6–1.1% compared with 3–5% of total DPD deficiency [15,18]. Therefore, some researchers have stated that DPYD genotyping and DPD phenotyping tests should be integrated in a two-step screening strategy [15,19].

## 4 Conclusions

The most important message from this case report is that, had we diagnosed the DPD deficiency in our patient, we could have used other treatment options. Thus, screening for DPD deficiency should be performed prior to 5-FU administration considering the wide use of 5-FU chemotherapy and relatively high frequency of its toxicity.

## Conflicts of interest

The authors declare no conflicts of interest.

## Funding

This research did not receive any specific grant from funding agencies in the public, commercial, or not-for-profit sectors.

## Ethical approval

Consent was obtained from the family of the patient. The study of a case report is exempt from ethnical approval in my institution.

## Consent

Signed consent was obtained from the family of the patient.

## Author contribution

Fumiya Inoue: Writing and case report design.

Takuya Yano: Writing and case report design, checking for accuracy.

Masahiro Nakahara: Determining the treatment plan and revising the manuscript.

Hiroshi Okuda: Helping to draft the manuscript.

Hironobu Amano: Determining the treatment plan and revised the manuscript.

Shuji Yonehara: Advising about pathology.

Toshio Noriyuki: Determining the treatment plan and revised the manuscript.

## Registration of research studies

Not applicable. This case report involved no patient recruitment.

## Guarantor

Takuya Yano.

E-mail: yano-tuk@umin.ac.jp.

## Provenance and peer review

Not commissioned, externally peer-reviewed.
